# Carbon Nanodots for On Demand Chemophotothermal Therapy Combination to Elicit Necroptosis: Overcoming Apoptosis Resistance in Breast Cancer Cell Lines

**DOI:** 10.3390/cancers12113114

**Published:** 2020-10-25

**Authors:** Aldo Nicosia, Gennara Cavallaro, Salvatore Costa, Mara Andrea Utzeri, Angela Cuttitta, Gaetano Giammona, Nicolò Mauro

**Affiliations:** 1 Institute for Biomedical Research and Innovation—National Research Council (IRIB-CNR), 90146 Palermo, Italy; aldo.nicosia@cnr.it; 2 Laboratory of Biocompatible Polymers, Department of “Science e Tecnologie Biologiche, Chimiche e Farmaceutiche” (STEBICEF), University of Palermo, 90123 Palermo, Italy; gennara.cavallaro@unipa.it (G.C.); maraandrea.utzeri@unipa.it (M.A.U.); gaetano.giammona@unipa.it (G.G.); 3 Department of “Scienze e Tecnologie Biologiche, Chimiche e Farmaceutiche” (STEBICEF), University of Palermo, 90128 Palermo, Italy; salvatore.costa@unipa.it; 4 Institute for studies on the Mediterranean-National Research Council- (ISMED-CNR), Detached Unit of Palermo, 90145 Palermo, Italy; angela.cuttitta@cnr.it; 5 eFondazione Umberto Veronesi, 20122 Milano, Italy

**Keywords:** carbon nanodots, theranostics, necroptosis, anticancer phototherapy, irinotecan, breast cancer, gene expression analyses

## Abstract

**Simple Summary:**

Carbon nanodots (CDs) are considered a versatile family of fluorescent, near infrared (NIR) active, and bioeliminable nanoparticles. Accordingly, the CDs application in photothermal therapy and theranostics increased. Problems limiting their use arise from the heterogeneity of most CDs and the lack of exhaustive information on their nanotoxicity at cellular and molecular levels. The lack of these data is often quite dramatic and causes substantial loss of translational value. To overcome this, we developed biocompatible homogenous CDs with a well-known structure as well as efficient red fluorescence and NIR photothermal conversion. The controlled photothermal effect and the on-demand release of the irinotecan successfully kill breast cancer cell lines in absence of relevant cell stress after internalization. We believe that these results provide insights to advance the field with significant impact, paving the way for the design of effective and safe nanomedicines for precision photothermal cancer therapies.

**Abstract:**

Background: Engineered luminescent carbon nanodots (CDs) are appealing nanomaterials for cancer image-guided photothermal therapy combining near infrared (NIR)–triggered hyperthermia, imaging, and drug delivery in a single platform for efficient killing of cancer cells. This approach would allow eliciting synergistic regulated cell death (RCD) routes such as necroptosis, targeting breast cancer cells refractory to apoptosis, thus overcoming drug resistance. Methods: We report the preparation of CDs bearing biotin as a targeting agent (CDs-PEG-BT), which are able to load high amounts of irinotecan (23.7%) to be released in a pulsed on-demand fashion. CDs-PEG-BT have narrow size distribution, stable red luminescence, and high photothermal conversion in the NIR region, allowing imaging of MDA-MB231 and MCF-7 cancer cells and killing them by photothermal and chemotherapeutic insults. Results: Cellular uptake, viability profiles, and RCD gene expression analyses provided insights about the observed biocompatibility of CDs-PEG-BT, indicating that necroptosis can be induced on-demand after the photothermal activation. Besides, photothermal activation of drug-loaded CDs-PEG-BT implies both necroptosis and apoptosis by the TNFα and RIPK1 pathway. Conclusions: The controlled activation of necroptosis and apoptosis by combining phototherapy and on-demand release of irinotecan is the hallmark of efficient anticancer response in refractory breast cancer cell lines in view of precision medicine applications.

## 1. Introduction

In the last decade, the necessity of more personalized, noninvasive, and efficient treatments in the fight against breast cancer has aroused considerable interest. Among the most promising tools proposed so far, the development of multifunctional nanotechnologies able to simultaneously act as photosensitizers and contrast agents (photothermal theranostic agents) have the highest therapeutic potential to tackle solid tumors such as breast cancer, overcoming the interindividual variability in therapeutic response [1,2]. Photothermal theranostic agents allow circumventing severe side-effects of conventional chemotherapy and multidrug resistance (MDR) by combining local and on-demand release of potent anticancer drugs and heat (hyperthermia). Moreover, they integrate targeted synergistic treatments and noninvasive imaging modalities (i.e., fluorescence imaging, MRI, etc.) in a single nanoplatform, thus enabling recognition, eradication, and monitoring of tumors [3,4,5]. This approach would allow at the same time early diagnosis, real-time monitoring, and selective therapeutic efficacy toward tumors (imaging-guided photothermal therapy—IG-PTT), contributing significantly to the ever-growing field of personalized medicine [6]. IG-PTT combining near-infrared (NIR)-triggered hyperthermia and fluorescence imaging (FL) has attracted wide attention due to its simplicity, noninvasiveness, safety, and remotely controllable properties [7]. Although hyperthermia, due to NIR-light energy conversion, induces cancer cell death through increasing temperature in the tumor site (over 41 °C), fluorescence imaging provides a detailed map of cancer cells to be eradicated [8,9]. In addition, clinical pieces of evidence indicate that local hyperthermia by IG-PTT not only induces cancer cell ablation but also enhances the antitumor effect of chemotherapy by rendering tumor cells more susceptible to anticancer drugs [10,11].

With the development of a theranostic, several nanomaterials such as gold nanoparticles, quantum dots, graphene oxide, and polymer-based nanoparticles have been proposed for cancer treatment and diagnosis [6,10,12,13,14,15,16,17,18,19]. However, the nanomaterials are considered as mere delivery agents and their inherent effects at the cellular level have so far attracted limited attention. By contrast, nanotoxicology of such nanomaterials and their potential inherent anticancer effects are topics of great interest for the design of real-world safe and effective nanomedicines useful in cancer IG-PTT.

In the last years, nanomedicine research is focusing on the effects of nanomaterials and therapeutic approaches, like hyperthermia, on the programmed regulated death pathway (RCD) such as necroptosis, autophagy, and apoptosis [20].

Chromosome condensation, nuclear fragmentation, membrane blebbing, and caspase-mediated cell death represent common apoptotic features. Canonical activation routes include the death receptor-mediated apoptosis pathway (extrinsic pathway) [21,22] and the mitochondrion-induced apoptosis pathway. In addition to the Tumour Necrosis Factor α/Fas Ligand (TNFα/FasL)-triggered extrinsic pathway leading to caspase 3 activation via caspase 8 [23], in the mitochondrion-dependent apoptosis pathway, the balance among proapoptotic and antiapoptotic factors regulates the caspase-9 activation, which, in turn, activates effector caspases [24,25]. However, resistance to apoptosis is responsible for both tumorigenesis and drug resistance. Thus, overcoming apoptosis resistance through the induction of nonapoptotic programmed cell death mechanisms represents an attractive therapeutic strategy.

The development of nanomaterials triggering the selective on-demand release of anticancer drugs in the site of action by NIR-light phototherapy offers the possibility of eliciting alternative regulated cell death (RCD) routes, overcoming apoptosis resistance, and maximizing therapeutic efficacy of treatments [20]. In this line, the route of necroptosis has been considered because of the strong potential for cancer treatment [20]. Necroptosis was discovered as a novel caspase-independent regulated necrotic cell death modality, considered as an alternative way of RCD to bypass apoptosis resistance [26,27]. Commonly, necroptosis occurs in the absence of caspases 3, 7, or the procaspase-8 activator FADD [28]. Generally, the necroptotic pathway is elicited through death receptors activation regulating the activity of RIPK1, RIPK3, and MLKL [27,29], which in turn regulate caspase 8 inhibition and promote the necrosome formation [27].

Scientific shreds of evidence have detected that PTT preferentially induces tumor cell death by necroptosis increasing the temperature well above the physiological range (43–49 °C) [30]. Besides, the potential role of necroptosis in cancer is much more interesting due to its capacity to induce strong adaptive immune response that play a crucial role in the regulation of cancerogenesis, metastasis, and cancer immunity. Moreover, the expression of key mediators of the necroptosis pathway is generally downregulated in different types of cancers, suggesting that cancer cells may also evade necroptosis to survive [20,26,27]. However, the induction of necroptosis pathways by nanomaterials and the role of necroptosis in cancer are still topics of controversy and need further investigations by the scientific community [20].

Among the nanomaterials with huge potential in IG-PTT, carbon nanodots (CDs) represent emerging luminescent photosensitizers exhibiting water-solubility, lower toxicity, and higher stability than other theranostic agents of the same family (e.g., carbon nanotubes and graphene oxide) [31]. In addition, they are ultrasmall (d < 5 nm) and can be bioeliminated after their biodistribution, thus avoiding the typical bioaccumulation issues of noble metal nanoparticles with similar theranostic potential such as gold nanoparticles [32]. CDs possess excellent photothermal conversion and fluorescence imaging properties with lower cytotoxicity than gold nanoparticles [6].

Hence, they exhibit a peculiar combination of optical and delivery properties that makes them promising platforms for biosensing, bioimaging, medical diagnosis, real-time tracking of drug release, and tumor therapy applications [33,34,35].

Red-emitting CDs have shown the most promising properties in cancer theranostic, since they emit light that can pass through biological tissues, without being absorbed, so as to give information on their localization in specific body districts. Along this line, we have demonstrated that good engineering of red-emitting CDs, in terms of synthetic approach and surface passivation with targeting agents such as biotin, allows controlling their size and surface characteristics as well as the optical properties and to confer cell-recognition capability towards breast cancer cells. Besides, the small and narrow size distribution of these CDs allows delivering a huge amount of bioactive molecules to be released after photothermal stimuli, thus making them multifunctional platforms that combine in a single tool selective chemotherapy and photothermal ablation [36].

This work aims to assess the biocompatibility of red-emitting carbon nanodots bearing biotin as a targeting agent, namely, CDs-PEG-BT, with potential in breast cancer IG-PTT. A deep analysis of their influence in triggering RCD machinery was made based on pathway-focused gene expression analysis correlating apoptosis, autophagy, and necroptosis cross-talking. In order to elicit selective RCD via necroptosis and overcome MDR on resistant breast cancer cell lines, namely, MCF-7 and MDA-MB231, we investigated the influence of photothermal-triggered on-demand release of irinotecan, used as an anticancer drug, combined with hyperthermia. A fine gene expression analysis and correlations with potential benefits owing to RDC mechanisms cross-talking are reported and proposed as a standard study for the development of new theranostic agents with good translational potential.

## 2. Results and Discussion

### 2.1. Rationale

Although nanomaterials are xenobiotic agents, once inside cells they might provoke cellular stress such as the production of reactive oxygen species (ROS), thus many of them would trigger regulated cell death (RCD) pathway [20]. To date, there are many opening questions about the ability of nanomedicines to elicit RCD, since cytotoxic effects of nanomaterials have been mainly investigated on the basis of cell viability and functionality assays such as mitochondrial activity and cell morphology. Here, we propose that a more detailed investigation of RCD triggered by nanomaterials such as carbon nanodots (CDs) is strictly necessary to understand their cytocompatibility and to target potential new anticancer effects exploitable in the design of safe and tailor-made nanomedicines for precision cancer medicine applications.

**Figure 1:**
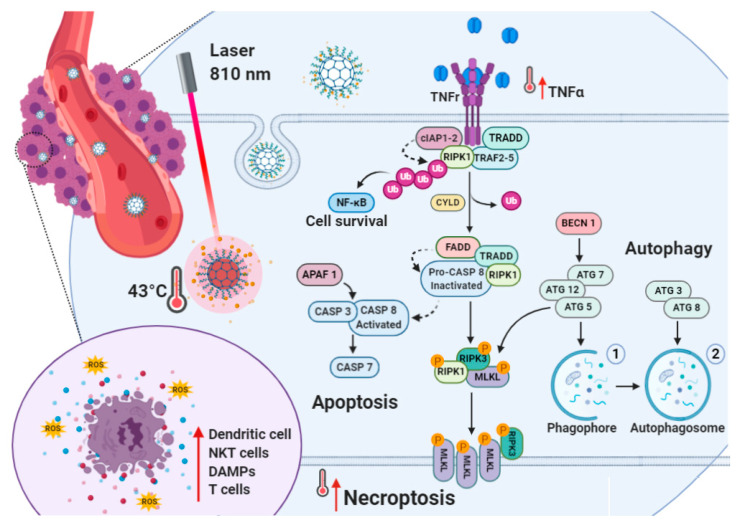
Near-infrared (NIR)-triggered photothermal action in MDA-MB-231 and MCF7 cell lines: the illustration shows irinotecan hydrochloride (IT) on-demand release from irinotecan-loaded biotinylated carbon nanodots (CDs-PEG-BT/IT) by means of hyperthermia. The cross-talks among apoptosis, autophagy and necroptosis are showed. After internalization, CDs-PEG-BT/IT act as nanoheaters under NIR irradiation promoting massive irinotecan release and generating hyperthermia so as to induce cell death. The increased expression of several proapoptotic factors, including Tumour Necrosis Factor α (TNFα), caspase 3 (CASP3), caspase 7 (CASP7), and caspase 9 (CASP9), acted to trigger cell death via apoptosis in MDA-MB-231. Additionally, the simultaneous upregulation of autophagic-related genes (ATG12, ATG3, and BECN1) and necroptosis marker collaborating network suggests the existence of simultaneous death mechanisms reinforcing the antitumor effect. In MCF7 cells with increased apoptotic threshold because of the absence of functional CASP3 and downregulation of APAF1, CASP7, and CASP9, CDs-PEG-BT/IT promote the upregulation of TNFα/RIPK1-dependent pathway, thus overcoming apoptosis resistance through the necroptotic pathway (red arrow).

It is well known that apoptosis resistance represents an obstacle leading to chemotherapy failure during cancer treatment [37]. As apoptosis is impaired in cancer cells, nanomedicines capable of inducing necroptosis would have important therapeutic benefits in patients (Figure 1). This is because necroptosis is a caspase-independent regulated necrotic cell death, mainly initiated by TNFα and RIPK1 pathways, that serves as an alternative route of RCD overcoming apoptosis resistance and might amplify antitumor immunity in cancer therapy (Figure 1) [20,38].

In fact, despite the role of necroptosis being debated, the expression of key regulators of the necroptotic pathway is generally downregulated in cancer cells. This is because necroptosis may provoke adaptive immune responses useful against tumor progression. We suggest that the on-demand delivery of proapoptotic anticancer drugs (e.g., irinotecan) via NIR light-induced hyperthermia through carbon nanodots may trigger both apoptosis and necroptosis, thus limiting the adaptive response of breast cancer cells (Figure 1). In fact, in previous work, we demonstrated that the combination of irinotecan with photothermal therapy by biotinylated carbon nanodots (CDs-PEG-BT) allows selective eradication of multicellular cancer organoids more efficiently if compared with the free drug alone [36].

It seems that, apart from imparting a detectable red fluorescence (quantum yield, QY % > 4) useful in imaging applications, CDs-PEG-BT provide eradication of 3D organoids after the exposure with a NIR laser at low power density. In the present study, we try to test the assumption that both apoptosis and necroptosis are involved in the enhanced anticancer effect observed for the irinotecan-loaded CDs-PEG-BT on two breast cancer cell lines (MDA-MB-231 and MCF7). These are phenotypically and metabolically different breast cancer cell lines, i.e., the triple-negative MDA-MB-231 and the estrogen-receptor-positive and caspase-3-deficient MCF7 [39,40]. In this scenario, the route of necroptosis has been considered of the huge potential for cancer treatment irrespective of hormone dependence or caspase-3 status. In particular, this investigation reports the effect of heating combined with irinotecan treatment in the induced cell death by means of RCD. The interaction of carbon nanodots and irinotecan in the development of necroptosis is established and proposed to design successful and safe nanotheranostic agents for the image-guided photothermal treatment of resistant breast cancers.

### 2.2. Preparation and Physicochemical Characterization of the Carbon Nanodots (CDs-PEG-BT)

Carbon nanodots (CDs) with controlled size distribution and surface are normally prepared by solvothermal reactions of small molecules such as multifunctional amines or amides in combination with multifunctional acids, followed by chromatography. Because of the heterogeneous nature of crude CDs containing small molecules as well as amorphous nanoparticles, their use in medical applications is precluded. Therefore, the use of chromatography for preparing pure CDs with selected size distribution and surface functional groups seems both facile and appealing. Here, as previously described, the use of size exclusion chromatography (SEC) allows isolation of the most red-luminescent CDs fraction capable of acting as a nanoheater in the NIR region [36]. These CDs have a QY of 4% and were used as red-emitting core of 1.5 ± 0.2 nm in diameter to prepare PEGylated CDs carrying biotin as side pendants. Surface functionalization of CDs was attained by amide coupling between carboxyl groups of CDs and the amine group of semitelechelic PEG chains carrying an amine group at one end-chain and an alkyne moiety at the other one (NH2-PEG_2000_-CC), and employing EDC and NHS as activating agents. After this step, the alkyne function on the PEGylated CDs surface was derivatized with azide-biotin chains by Cu(I)-catalyzed 1,3-dipolar cycloaddition [36].

**Figure 2:**
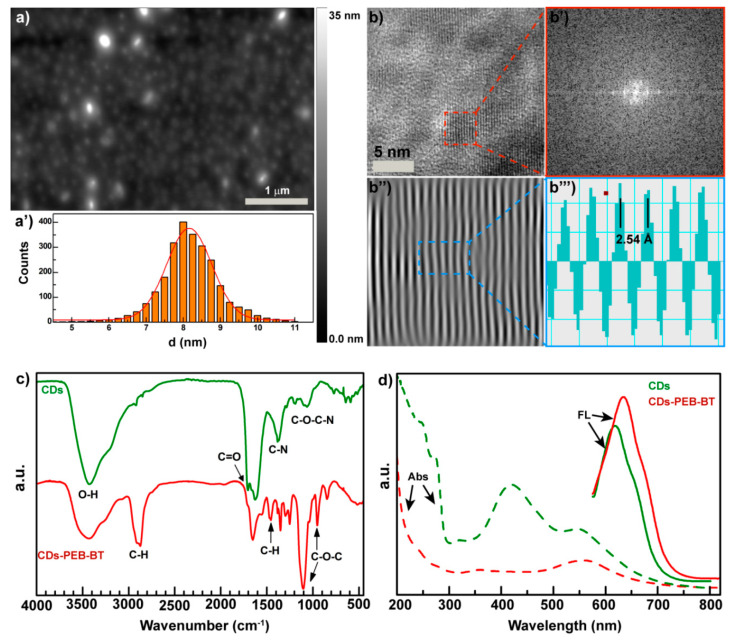
Physicochemical characterization of the CDs-PEG-BT. Atomic force microscopy (AFM micrograph (**a**) and size distribution (**a’**) of the CDs-PEG-BT. HRTEM micrograph (**b**), fast Fourier transform (FFT) (**b’**), inverse fast Fourier transform (IFFT) (**b’’**), and d-spacing calculation (**b’’’**) of the CDs-PEG-BT. FTIR spectra of the CDs-PEG-BT compared with the bare CDs (**c**). Absorption and emission spectra excited at 550 nm of an aqueous dispersion of CDs-PEG-BT and the bare CDs (**d**).

Atomic force microscopy (AFM) of the biotinylated CDs, henceforth named “CDs-PEG-BT,” shows isolated nanodots with very narrow size distribution (Figure 2a). Indeed, size distribution obtained from AFM micrographs provides evidence that CDs-PEG-BT of 8.2 ± 0.5 nm in diameter were obtained (Figure 2a’). The increased CDs shell is a good evidence that PEGylation occurred at the CDs’ surface by means of stable covalent bonds. Besides, high-resolution transmission electron microscopy (HRTEM) reported in Figure 2b shows that CDs core persists after surface functionalization processes, indicating the formation of a core–shell structure. The analysis of the fast Fourier transform (FFT) and the inverse fast Fourier transform (IFFT) of the HRTEM micrograph allows calculating the d-spacing of the CDs core (Figure 2b’,b’’’). No significant changes in the CDs lattice were observed, implying that only surface groups of CDs were involved in the reaction. The covalent reaction between carboxylic groups of CDs and the amine end-chain of PEG-BT chains is confirmed by FTIR analysis reported in Figure 2c. The amount of carboxyl groups observed at 1720 cm^−1^ decrease in the CDs-PEG-BT sample if compared with the parent CDs, accompanied to an increase in the amide I and amide II bands at 1670 and 1590 cm^−1^, respectively. In addition, the typical mode of vibrations of PEG chains appeared in the CDs-PEG-BT spectrum at 985, 1104, 1483, and 2900 cm^−1^. These data indicate that the bare CDs contained several COOH reactive groups amenable to conversion to amide via coupling reactions with NH2-PEG_2000_-BT chains.

Here, the CDs core was designed to impart to the nanomedicine theranostic properties, combining self-tracking features in fluorescence imaging applications and photothermal ability. The bare CDs sample shows several absorption bands in the UV/VIS spectrum, whereas PEGylation implies a simpler spectrum with two main absorption bands roughly at 350 and 560 nm (Figure 2d). As a consequence, surface covalent functionalization of the CDs-PEG-BT yield isolated red emission at 640 nm with a QY of 4.5%, significantly shifted if compared with the pristine CDs core (Figure 2d). These findings corroborate the hypothesis of the involvement of the CDs surface in the coupling reaction with PEG chains since the origin of the red shift upon functionalization can be ascribed to changes in the energy level of the surface acceptor state at the crystalline surface of the CDs core [26,29].

### 2.3. CDs-PEG-BT Allow High Irinotecan Loading and On-Demand Light-Triggered Drug Release

**Figure 3:**
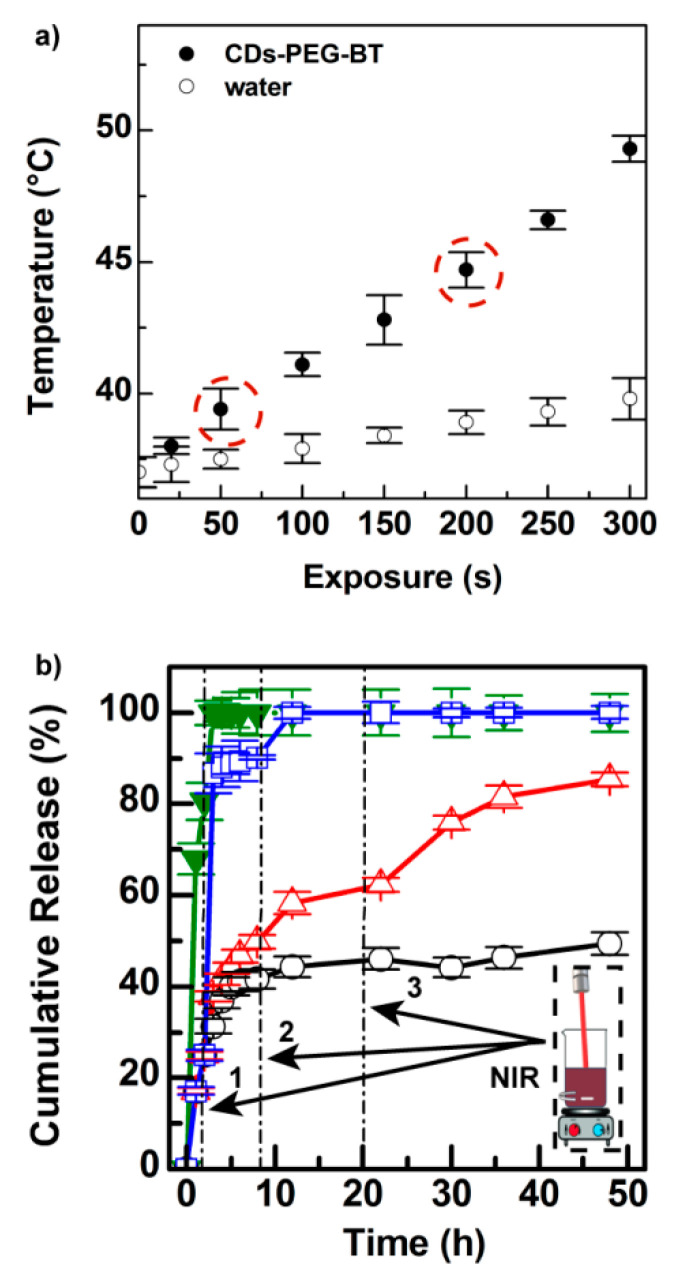
Photothermal kinetics of the CDs-PEG-BT in PBS pH 7.4 (0.5 mg mL^−1^) (**solid symbol**) compared with pure phosphate buffered saline (PBS) (open symbol) (**a**). Drug release profiles of CDs-PEG-BT/IT in PBS pH 7.4 compared with the diffusion profile of the free IT (green line) at the equivalent concentration (**b**): without 810 nm laser exposure (**black line**), after three cycles of NIR exposure for either 50 s (**red line**) or 200 s (**blue line**) at low power density (2 W cm^−2^). Release profiles of CDs-PEG-BT/IT in PBS pH 7.4 after three cycles of 810 nm laser exposure for either 50 or 200 s (2 W cm^−2^).

The light-triggered photothermal effect of CDs-PEG-BT is reported in Figure 3a. The red-emitting CDs core possesses good photothermal conversion (25.8%), whereas the existence of possible bias in NIR photothermal conversion due to the presence of PEG shell was ruled out since the outstanding increase in temperature was observed under 810 nm laser diode irradiation (Figure 3a). 

In particular, the temperature of an aqueous dispersion of CDs-PEG-BT (0.5 mg mL^−1^) increased up to 48 °C after 300 s of laser exposure at low power density (2 W cm^−2^). Moreover, pure water undergoes only a temperature increase of about 2.5 °C under the same conditions. It is interesting to notice that CDs-PEG-BT possesses a tunable photothermal effect as a function of the NIR-light exposure time and power, which may be of help to control hyperthermia (41–43 °C) during the anticancer treatment, thus avoiding damages in the surrounding healthy tissues. The photothermal effect of CDs-PEG-BT, coupled with its good emission in the biologically transparent window (red region), makes them potential candidates as theranostic agents in image-guided photothermal therapy (IG-PTT).

Here, the IG-PTT approach is proposed to provoke local hyperthermia inside tumors in order to induce both heat shock and massive drug release in situ. CDs core possesses anionic charge residues (1.51 me g^−1^) after surface passivation under the same conditions adopted in the present study and they would allow loading cationic drugs such as irinotecan (IT) [36]. In fact, the PEG shell and carboxyl groups at the CDs surface were employed to load IT inside CDs-PEG-BT by simply sonicating a suspension of CDs-PEG-BT in the presence of IT. The amount of drug loaded in CDs-PEG-BT/IT, measured by HPLC, reaches a maximum of 23.7% with an adsorption efficiency of 30% on a weight basis. The extremely high drug loading can be explained by the huge specific surface area displayed by these very small nanodots. 

The ability of the CDs-PEG-BT/IT of releasing the drug in an on-demand fashion was established by measuring the amount of drug released at equilibrium with and without repeated NIR-light stimuli at low power. Figure 3b shows the release kinetic of a dispersion of CDs-PEG-BT/IT in PBS pH 7.4 after three cycles of 810 nm laser exposure for either 50 or 200 s (2 W cm^−2^). For comparison purposes, the release profile of free IT and untreated CDs-PEG-BT/IT is reported as well. The untreated CDs-PEG-BT/IT retains the drug payload over time releasing about 54% of IT after 48 h of incubation. The same experiment carried out under 50 s of NIR laser exposure after 2, 8, and 20 h of incubation yield 88% of drug release after 48 h. In particular, a sharp release of IT is observed after applying the NIR laser, with a jump of about 20% in terms of released payload. A much more remarkable effect is observed under 200 s of NIR laser exposure, where 90% of drug release occurred after the cycle to reach 100% release after the second one (8 h) (Figure 3b). On the whole, release experiments imply that hyperthermia triggers IT release on-demand with a controlled release profile as a function of the laser power and exposure time. This is a particular interesting property in a real-world application since the personalization of the therapeutic approach is required to provide high efficacy and low target toxicity.

### 2.4. Biological Characterization

The in vitro cytocompatibility of CDs-PEG-BT was investigated on MDA-MD-231 and MCF7 cell lines. To demonstrate that CDs-PEG-BT do not provoke significant cellular stress after cell internalization, both functionality and gene expression assays were performed.

**Figure 4:**
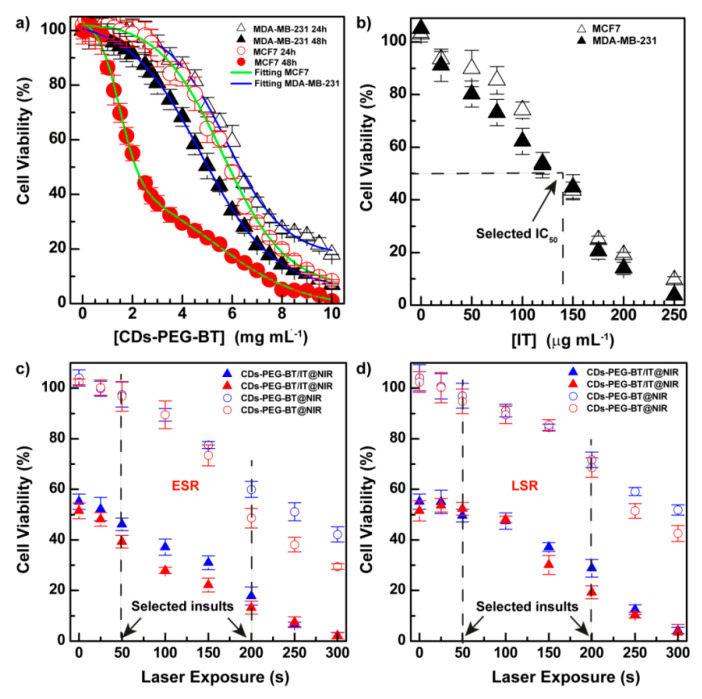
3-(4,5-dimethylthiazol-2-yl)-5-(3-carboxymethoxyphenyl)-2-(4-sulfophenyl)-2H-tetrazolium (MTS) assay on MCF7 and MDA-MB-231 cell lines. Cytocompatibility of CDs-PEG-BT on MCF7 and MDA-MB-231 cells after 24 and 48 h incubation. (**a**) dose–response or bi-dose–response fitting (R^2^ > 0.997) is reported in green and blue for MCF7 and MDA-MB-231 cells, respectively. Anticancer effect of CDs-PEG-BT/IT as function of equivalent amount of drug; (**b**) NIR-triggered anticancer photothermal effect of either the CDs-PEG-BT/IT and equivalent amount of the bare CDs-PEG-BT on MCF7 (red) and MDA-MB-231 (blue) cells at the IC50 calculated in Figure b (IT = 140 µg mL^−1^ ≡ CDs-PEG-BT = 590 µg mL^−1^) and low power density (2 W cm^−2^); (**c**–**d**): early stage response (ESR) is evaluated before the treatment (**c**), whereas long stage response (LSR) is evaluated 20 h after the photothermal insults (**d**). Data shown as mean ± s.e.m. (*n* = 3, two independent replicates).

First, cell viability was investigated by 3-(4,5-dimethylthiazol-2-yl)-5-(3-carboxymethoxyphenyl)-2-(4-sulfophenyl)-2H-tetrazolium (MTS) assay incubating CDs-PEG-BT for 24 and 48 h at a very high concentration (up to 10 mg mL^−1^). Results are reported in Figure 4a, whose cell viability profiles show that CDs-PEG-BT do not perturb mitochondrial activities at the concentration usually employed for in vitro and in vivo studies (1 mg mL^−1^). However, at concentrations higher than 1 mg mL^−1^, especially after 48 h of incubation, a different behavior of the two cell lines used can be observed. In particular, cell toxicity follows a dose–response trend for MDA-MD-231 as bimodal dose–response curves are observed for MCF7 cells. Besides, a remarkable time-dependent cytotoxic effect is observed at high dosage for the MCF7 cell line. As described below, no significant perturbation of the gene expression of RCD pathways was revealed under CDs-PEG-BT dosage (0.5 mg mL^−1^).

The in vitro anticancer effect of CDs-PEG-BT/IT or equivalent amount of free IT was carried out in cultures of MCF7 (estrogen receptor positive, ER2+; biotin receptor positive, BR+++) and MDA-MB-231 (triple-negative, BR++), two human breast cancer (HBC) cell lines overexpressing different amounts of BR. They also represent cancers with distinctive inclination to invade premetastatic niche and hence can be used as models to perform a comparative study on the anticancer effect of our theranostic agent [41].

As shown in Figure 4b, the cell viability of both cells decreased in a dose-dependent way at similar potency (IC_50_ ≈ 140 mg mL^−1^). The IC_50_ value observed was selected to perform photothermal experiments on both cell lines. In particular, the early stage response (ESR) of cancer cells toward NIR insults was established after irradiating cells with an 810 nm laser diode laser and measuring cell viability after 30 min of postincubation (Figure 4c). However, the long stage response (LSR) was measured after 20 h of postincubation from photothermal treatments (Figure 4d). This is to show how cells can respond to photothermal stress after a few minutes and after a long time. Figure 4c shows that CDs-PEG-BT at equivalent concentration of the IC_50_ observed in Figure 3b (590 µg mL^−1^) shows a decrease in cell viability up to 70% after 300 s of irradiation. Moreover, as a rule, photothermal insults are more dangerous for MCF7 cells. The effect of the combination between phototherapy and on-demand release of IT is outstanding comparing the curves on the bottom (Figure 4c), where cell viability reaches 1.8% at the maximum dose of phototherapy (300 s). Thus, the ESR to the combination between apoptotic effects of IT and photothermal effects of CDs-PEG-BT suggests activation of efficient cell death mechanisms. As expected, a similar dose-dependent trend was observed for the LSR experiments, but the photothermal effect registered at low dosage appears much more attenuated (Figure 4d). This mainly relies on the reintegration of cell growth pathways just after photothermal insults in resistant cells, but only if the inflicted damages are insufficient to trigger RCD phenomena. This is particularly self-evident until 100 s of irradiation at 2 W cm^−2^.

**Figure 5:**
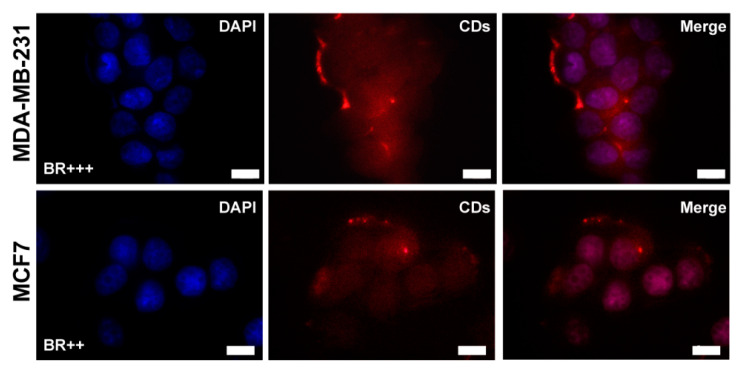
Cell uptake by fluorescence microscopy on MDA-MB-231 and MCF7 cell lines incubated for 4 h with CDs-PEG-BT (100 µg mL^−1^). Scale bar: 10 µm (100× magnification). Nuclei (DAPI channel—**blue**), CDs-PEG-BT (Txr channel—**red**).

The ability of CDs-PEG-BT/IT to enter cancer cells by biotin receptors (BR) and act as imaging agent in FL imaging applications was established by fluorescence microscopy on both cell lines considered and after 4 h of incubation. Figure 5 shows that the nanosystem can efficiently enter both MCF7 and MDA-MB-231 and diffuse into cells. A clearly visible red fluorescence ascribable to CDs-PEG-BT is detected inside nuclei, the cytosol, and inside endosomes.

It seems that both cell lines have many vesicles close to cell membranes attributable to CDs-PEG-BT localized inside endosomes (red FL). This is in agreement with the known huge expression of BR on the cell membrane of both cell lines. It might be noticed that CDs-PEG-BT have a diameter of about 8 nm, which permits their diffusion across nuclear pores (0.6–10 nm pore size) [42]. Besides, Figure 5 displays clear and stable self-tracking abilities of CDs-PEG-BT/IT owing to the excellent red fluorescence exhibited to the CDs core.

#### Gene Expression Studies on MDA-MB-231 and MCF7 Cell Lines

It is widely accepted that irinotecan inhibits the DNA-Topo-I complex, altering the transcriptional expression of proapoptotic factors and surviving genes in cancer cells, and induces RCD by apoptosis [43,44,45]. Moreover, we have previously shown that biotin-decorated CDs act as NIR-activated nanoheaters able to induce local hyperthermia thus efficiently impairing viability [36]. With the aim of confirming CDs-PEG-BT cytocompatibility at molecular level and gain further insights on cell death mechanisms triggered by CDs-PEG-BT and IT-loaded CDs-PEG-BT upon NIR irradiation, the differential mRNA expression levels of 31 genes involved in the RCD and necrosis pathways were assessed in MDA-MB-231 and MCF7 breast cancer cell lines. MDA-MB-231 and MCF7 were selected since they possess different genetic background mainly consisting in the different status of *P53* and *CASP3* conferring certain death resistance to MCF7 [39,46]. Interestingly, the molecular mechanisms of various types of cell death are distinct but also overlapping thus members ascribed to a certain pathway also play a pivot role in different death mechanisms [29,47,48]. Taking into account this crosstalk, we chose 14 genes recognized to exert a functional role in cell apoptosis including pro- and antiapoptotic genes (*APAF1, BAX, BCL2L11, CASP3, CASP7, CASP9, CYLD, FAS, TNFα, BCL2, BCL2A1, BCL2L1*, *CASP2,* and *MCL1*). Similarly, genes encoding members involved in autophagic programmed cell death (*ATG12, ATG3, ATG5, ATG7, BECN1, MAP1LC3A,* and *PIK3C3*) were transcriptionally analyzed. Transcriptional changes in genes with a recognized pivot role in the necrotic/necroptosis pathway were also profiled; these include *BMF, COMMD4, EIF5B, GALNT5 PARP1, PARP2, RAB25, TMEM57, RIPK1,* and *SLC25A4*.

**Figure 6:**
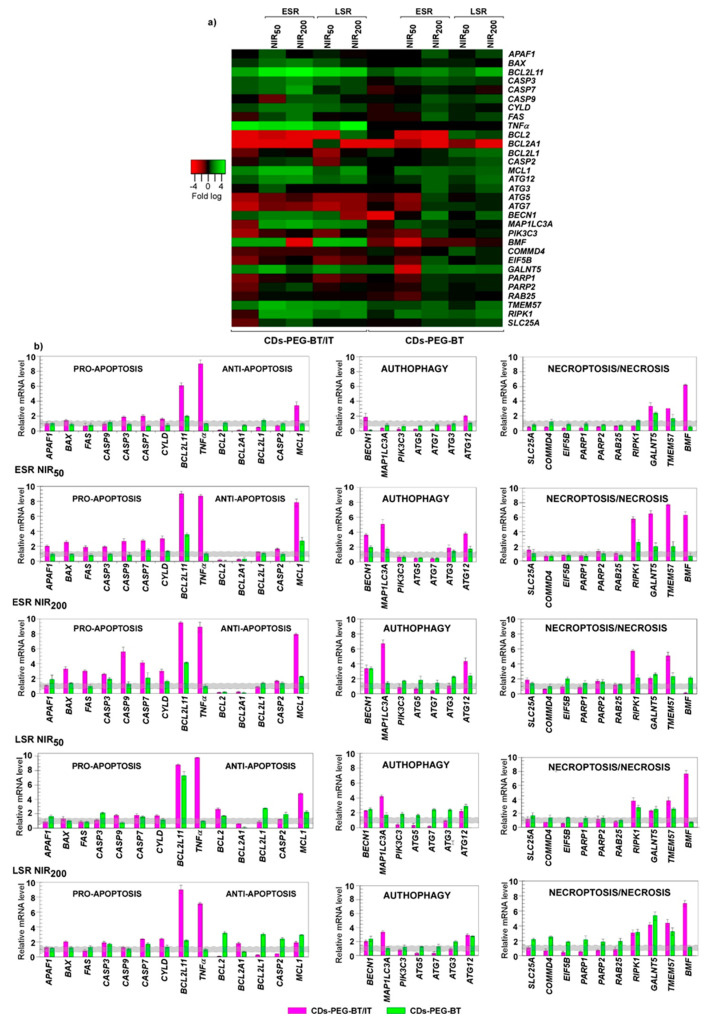
Cell death pathway focused gene expression analyses in MDA-MB-231. (**a**) Heat map representation of the mean-centered data of RT-qPCR results showing the mRNA levels of analyzed genes in presence of CDs-PEG-BT or CDs-PEG-BT/IT with respect to *18S*, *GAPDH,* and *ACTB*. Gene expression values are colored from red (low) to green (high). Fold-changes in log (2)-transformed values are represented. (**b**) Bar plot of the pathway focused gene expression profiling. The early stage response (ESR) and long stage response (LSR) measured in response to 50 and 200 s of photothermal insults (NIR_50_ and NIR_200_) are showed. Values beyond the gray zone were considered statistically significant at *p* ≤ 0.05. Statistical analyses by Student’s *t*-tests are reported in Table S2.

A comparison between cells treated with CDs-PEG-BT and unexposed MDA-MB-231 revealed a similar transcriptional profile. Of the 31 cell death pathway-focused genes, only 2 showed a 2-fold difference in gene expression in CDs-PEG-BT-treated cells with negligible effects, if any, on the activation of a death response (Figure 6a,b). As expected, MDA-MB-231 cells exposed to CDs-PEG-BT/IT showed different gene expression pattern when compared with the control. Among the gene set analyzed, we found at least 1.5-fold difference in gene expression of 23 genes between cells treated with CDs-PEG-BT/IT and controls. Upregulation was observed in 11 genes, whereas 12 genes underwent downregulation. Cells treated with CDs-PEG-BT/IT showed a number of genes associated with apoptotic cell death exhibiting significant differential expression. In particular, among 11 upregulated genes, 5 encode for proapoptotic members and include *BCL2L11*, *CASP3*, *CASP7*, *CYLD*, and *TNF*α. Additionally, a reduction in the gene expression levels of prosurvival factors as *BCL2*, *BCL2A1,* and *BCL2L1* occurred. Conversely, the mRNA levels of autophagic- and necrotic-related genes were reduced since the majority of downregulated genes falls in these pathways. These results suggest the activation of the apoptotic cell death pathway in MDA-MB-231 exposed to CDs-PEG-BT/IT, which is responsible for the reduction in cell viability observed in Figure 4b. Because the cell response to photothermal insults also depends on the amount of energy absorbed by internalized CDs-PEG-BT and CDs-PEG-BT/IT nanoparticles, these effects were evaluated in MDA-MB-231 cells treated for 50 and 200 s at 2 W cm^−2^ laser irradiation. At ESR, a few changes in the expression profiles occurred in response to 50-s laser treatment for the cells exposed to CDs-PEG-BT (Figure 6a,b), without hinting at the activation of death response altering MDA-MB-231 viability (Figure 4c). The reverse transcription -quantitative PCR (RT-qPCR )analyses also showed that in response to 200-s laser treatment the mRNA levels of 10 genes were at least 1.5-fold higher. In particular, it emerges a concurrent upregulation of apoptotic- (*BCL2L11*, and *CASP3*), autophagic- (*ATG12*, *ATG3*, *BECN1,* and *MAP1LC3A*), and necroptotic (*TMEM57*, *BMF*, *GALNT5,* and *RIPK1*)-related genes (Figure 6a,b). It is well recognized that different cell death mechanisms may simultaneously occur in cells exposed to certain stimuli [29]. Thus, considering the magnitude of the measured upregulations, a concurrent and slight activation of different cell-death programs could be hypothesized under this condition so as to impair cell viability (Figure 4c). Additionally, mutually transforming commitment toward a specific pathway could be also supposed because of the interconnection of the herein analyzed members in death signaling pathways [49,50,51].

The ESR with 50 s laser treatment of MDA-MB-231 cells incubated with CDs-PEG-BT/IT resulted in an upregulation of 19 genes at least with 1.7-fold difference comparing to controls. It should be noticed that the co-occurrence of irinotecan-related DNA damages and the hyperthermia treatment reinforced the upregulation of *BCL2L11*, *CASP3*, *CASP7*, *CYLD,* and *TNFα* (Figure 6a,b). Besides, the combination of hyperthermia and IT release implies a significant increase in the transcriptional rate of the proapoptotic genes *BAX*, *FAS*, *APAF1,* and *CASP9*, in addition to a huge downregulation of the antiapoptotic genes *BCL2* and *BCL2A1,* thus stimulating the activation of a cascade of proteolytic events leading to apoptosis. In this context, it is interesting to note that under this experimental asset, the brief NIR laser exposure of cells causes a further reduction in the cell viability of about 10% if compared with the nonirradiated ones. We also observed the upregulation of several genes involved in autophagic- (*ATG12*, *ATG3*, *BECN1* and *MAP1LC3A*) and necroptotic (*TMEM57*, *BMF*, *GALNT5* and *RIPK1*)-related pathways (Figure 6a,b). Because a similar pattern was observed in MDA-MB-231 incubated with CDs-PEG-BT, it is likely to represent a transcriptional signature of cell response to NIR-triggered local hyperthermia. It has been shown that factors previously known to regulate a specific pathway were recently recognized to play pivot role in different cell death mechanisms. Among them, *MCL1*, a *BCL2* antiapoptotic homolog, has been described as regulators in the balance between apoptosis and autophagy [52,53]. Herein, qPCR analyses also revealed that among *BCL2* antiapoptotic homologues, exclusively, *MCL1* showed a huge transcriptional increase; thus, it is tempting to speculate on additional levels regulating the MDA-MB-231 fate. In this scenario, the concurrent existing RCD pathways owing to photothermal treatments provide efficient input to avoid drug-resistance phenomena. 

The 200 s laser treatment provides overlapping expression profiles with further increase in apoptosis-related genes, namely, *BAX*, *BCL2L11*, *CASP3*, *CASP7*, *FAS*, *TNFα,* and the same downregulation of prosurvival genes discussed above *(BCL2* and *BCL2A1)*. Therefore, it is reasonable to argue that the higher photothermal effect (Figure 3a: T = 44.5 °C), together with a massive on-demand release of IT (Figure 3c) provides irreversible stimuli promoting cell death via activation of both apoptosis and necroptosis, thus enhancing the antitumor effect on MDA-MB-231 (cell viability lower than 20%).

We also investigated the long stage response (LSR) of cells treated with the proposed nanosystem so as to assess the maintenance of these responses after a postincubation period. To this purpose, MDA-MB-231 cells were incubated for 4 h with either CDs-PEG-BT or equivalent amount of CDs-PEG-BT/IT; then, we applied NIR laser exposures as described above, and the pathway focused gene expression analyses were profiled after 20 h of postrecovery (Figure 6a,b). For the cells treated with the virgin CDs-PEG-BT, the LSR observed after 50 s of laser treatment was similar to that occurred in the ESR. In particular, the slight increase in the mRNA levels of 2 proapoptotic factors (*BCL2L11* and *CASP3*) resulted counteracted by the upregulation of prosurvival genes (*BCL2*, *BCL2L1*, *CASP2,* and *MCL1*), suggesting that no cues triggering apoptosis occurred in such cells. Figure 6a,b shows a slight upregulation of some autophagic- and necroptotic-related factors, including *ATG12*, *ATG7*, *COMMD4*, *GALNT5,* and *RIPK1*, suggesting a certain rule of both RCD in the LSR after minor laser applications. The 200 s laser treatment resembled those observed in the ESR study. Indeed, the upregulation of *BCL2L11*, *CASP3,* and *CASP7* appeared to fail in the activation of apoptosis, since the increased mRNA levels of several prosurvival genes (*BCL2*, *BCL2L1*, *MCL1* and *CASP2*) acted to neutralize them. However, the maintenance of simultaneous induction of autophagic- and necroptotic-related members are likely to retain some effects on cell viability (Figure 4c and Figure 6a,b). By contrast, a different expression pattern was revealed in LSR of MDA-MB-231 incubated with CDs-PEG-BT/IT and exposed to the selected NIR laser exposures. Both the 50 and 200 s NIR irradiation provide an overall mRNA expression profiles similar to those observed in the ESR, but with qualifications. Not surprisingly, the upregulations provoked by hyperthermic treatments, namely, *ATG12*, *ATG3*, *BECN1*, *MAP1LC3A*, *TMEM57*, *BMF*, *GALNT5,* and *RIPK1*, were attenuated (Figure 6 a,b). Despite a general reduction in the amplitude of transcriptional response, RT-qPCR analysis revealed that the co-occurrence of IT-related DNA damages and NIR-induced hyperthermia maintains prosurvival genes as *BCL2* and *BCL2L1* strongly downregulated and sustain the upregulation of apoptosis- and necroptosis-related genes, ensuring a good antitumor effect even after cell recovery (Figure 4d and Figure 6a,b). As shown in Figure 1, the outstanding cross-talking between these two RCD pathways suggests that necroptosis supports the programmed cell death mechanisms normally triggered in MDA-MB-231 during IT exposure. 

**Figure 7:**
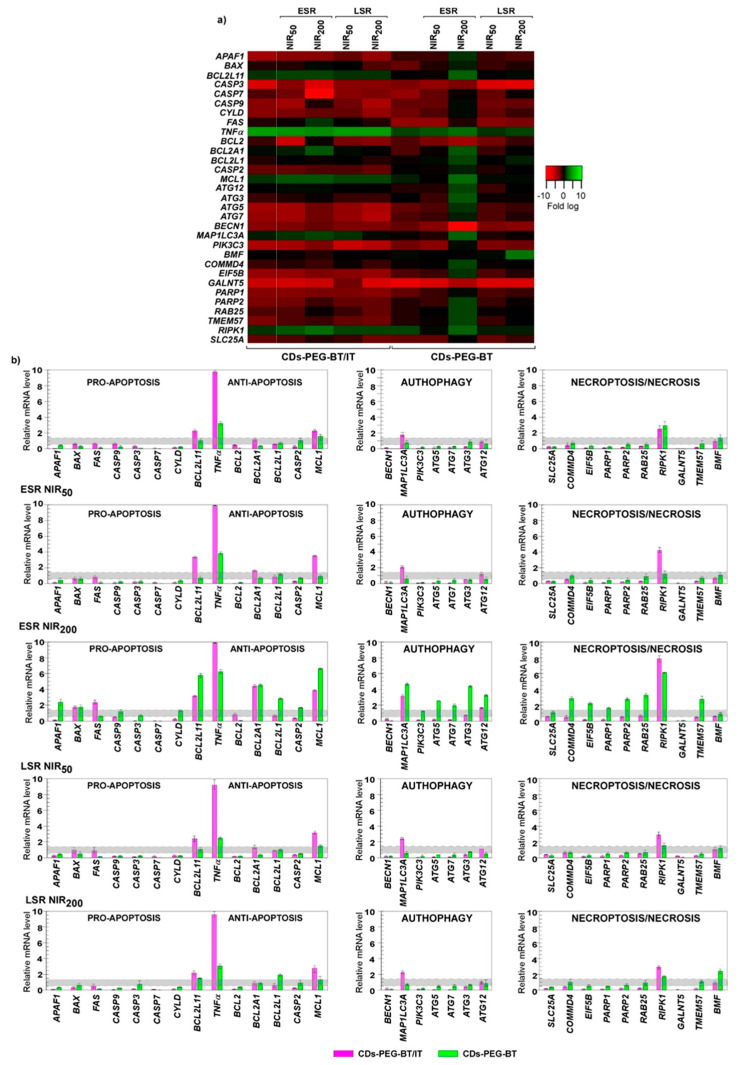
Cell death pathway focused gene expression analyses in MCF7. (**a**) Heat map representation of the mean-centered data of RT-qPCR results showing the mRNA levels of analyzed genes in presence of CDs-PEG-BT or CDs-PEG-BT/IT with respect to *18S*, *GAPDH,* and *ACTB*. Gene expression values are colored from red (low) to green (high). Fold-changes in log (2)-transformed values are represented. (**b**) Bar plot of the pathway focused gene expression profiling. The early stage response (ESR) and long stage response (LSR) measured in response to 50 and 200 s of photothermal insults (NIR_5_, and NIR_200_) are showed. Values beyond the gray zone were considered statistically significant at *p* ≤ 0.05. Statistical analyses by Student’s *t*-tests are reported in Table S3.

It is certainly worthy of remark that the transcriptional response of MCF7 upon CDs-PEG-BT exposure was quite different (Figure 7a,b). Indeed, 18 differentially expressed genes were measured, and, among them, the levels of 16 transcripts underwent downregulation, whereas two genes were upregulated. Even if such changes did not result in the activation of any RCD pathway at the low dose used. This different gene expression response would explain well the lower cytocompatibility observed in MCF7 at high dose and after chronic exposure (Figure 4a; IC_50_ = 2.1 and 4.9 mg mL^−1^ for the MCF7 and MDA-MB-231, respectively).

CDs-PEG-BT/IT stimulate a peculiar gene expression response in MCF7 cells. As 5 genes showed upregulation, 16 were downregulated for a total of 23 differentially regulated genes (Figure 7a,b). Upregulated genes include the proapoptotic members *BCL2L11* and, in particular, *TNFα*, which also is involved in triggering necroptosis (Figure 1). Because of the inactivation of crucial apoptotic effectors such as caspase-3 [39] and epigenetic events disabling P53 activity [46], MCF7 are refractory to apoptosis, thus the upregulation of *TNFα* in MCF7 can be associated to the control of necroptosis cross-talking (a caspase independent way; Figure 1). According with this assumption, not surprisingly, the *CASP3* appeared downregulated after the treatment with CDs-PEG-BT/IT at their IC_50_ value (Figure 4b). As a consequence, it is reasonable to assume that in absence of upregulation of antiapoptotic members, CDs-PEG-BT/IT provoke 50% cell death by releasing IT and slightly triggering caspase-independent cell death mechanisms in apoptotic-resistant cells. However, being collateral, this pathway was not enough effective to overcome drug resistance in MCF7 cells. 

On the whole, as for the nonirradiated cells, a feeble effect was observed on the gene expression profile of MCF7 cells treated with CDs-PEG-BT and 50 s NIR treatment at ESR (Figure 7a,b). The expression profile was mainly unchanged, whereas a reduction in the mRNA level of 10 genes was measured. According to cell viability data (Figure 4c; 92% cell viability), it is unlikely that the activation of death mechanisms results in altering MCF7 viability, which remains similar to the control. Different responses were detected in 200 s irradiated cells at ESR in which the prolonged NIR exposure resulted in the upregulation of 20 genes at least with 1.5-fold difference comparing to controls, whereas the others resulted unchanged or downregulated. Upregulated genes include *APAF1*, *BAX*, *BCL2L11,* and *TNF*α, which exert proapoptotic activity. However, also prosurvival members as *BCL2A1*, *BCL2L1*, *CASP2,* and *MCL1* were overexpressed, thus counteracting the activation of the apoptotic pathway. Interestingly, *ATG12*, *ATG3*, *ATG5*, *ATG7,* and *MAP1LC3A*, which usually act to promote autophagy-related cell death, and key factors involved in necroptosis signaling (*COMMD4*, *EIF5B*, *GALNT5*, *PARP1*, *RIPK1*, *RAB25*, *TMEM57*, *PARP2* and *CYLD)* were upregulated upon irradiation [54,55]. This scenario suggests a fine cross-talk among RCD machinery, which defines the cell fate upon a such strong insult [29,56]. It has been shown that the autophagic apparatus may act as a scaffold mediating necrosome assembly [57]. Therefore, it could be hypothesized that NIR irradiation of internalized CDs-PEG-BT in apoptosis refractory cells such as MCF7 promotes the activation of necroptotic pathways, via TNFα and RIPK1, using the autophagy machinery as scaffold for the efficient formation of the necrosome (Figure 1). This is responsible for 40% reduction in cell viability (Figure 4c).

Conversely, a different expression pattern was observed in response to NIR laser insults for the MCF7 cells treated with CDs-PEG-BT/IT. The 50 s laser insult caused an overall mRNA expression profiles resembling those observed in nonirradiated cells exposed to CDs-PEG-BT/IT, resulting in the upregulation of 6 genes and the reduction in the mRNA levels of 10 genes. In particular, RT-qPCR analysis revealed that the co-occurrence of irinotecan-related DNA damages and 50 s NIR irradiation enhanced the upregulation of proapoptotic members of *BCL2L11* and *TNFα* in addition to *RIPK1*, which has been measured in response to CDs-PEG-BT/IT alone. Thus, it appears that the photothermal stress additively acted to increase the cell death.

The 200 s laser insult exerted similar changes in the gene expression profile since mRNA levels of 9 genes were upregulated at least 1.5-fold and 17 transcripts were downregulated. Similarly to what occurred with MCF7 cells incubated with CDs-PEG-BT/IT at the IC_50_ value, in addition to the increased expression of *BAX* and *FAS*, the photothermal insult hugely reinforced *BCL2L11*, *TNFα,* and *RIPK1* transcriptional levels. However, in such experimental asset, the overall upregulation of antiapoptotic and autophagic members appeared mitigated when compared to MCF7 cells incubated with virgin CDs-PEG-BT, thus providing evidence that CDs-PEG-BT/IT exposure may preferentially trigger different signaling pathways or abrogate ones that have acted in response to single insult. 

It is known that necroptosis participates in cell death mechanism when caspase-dependent pathways are abrogated or in presence of an increased apoptotic threshold [27]. Similarly, it has been reported the activation of necroptosis in *APAF1*-deficient embryos, which are not able to promote caspases activation [58]. Taking this in mind, the measured downregulation of *APAF1* and *CASP3*-*7* and -*9,* associated with elevated mRNA levels of the main necroptotic actors (e.g., *TNFα* and *RIPK1*), provides evidence for the overcoming of MCF7 apoptosis resistance through activation of necroptotic pathway, which caused reduction in cell viability up to 15%. 

Particularly, noteworthy is the overlapping patterns of gene expression observed between cell response at ESR and LSR, even if the amount of gene overexpressed in response to the selected insults appeared attenuated (Figure 7a,b). This trend can be explained considering the recovery of cell growth after the postincubation period of 20 h. In particular, for MCF7 cells treated with CDs-PEG-BT, with and without NIR laser exposure, insults failed to produce a significant activation of RCD mechanisms, according to cell viability data (Figure 4d).

Remarked expression profiles, even if analogous, occurred in response to the 200 s laser insult. The prolonged photothermal insult caused the moderate upregulation of necroptotic regulators as TNFα, BMF, and RIPK1. Even though such members are recognized as the core of necroptotic signaling [47,59], it appears that the measured overexpression may not fulfill all the requirements for efficient activation of a cell death signaling. This is confirmed by the moderate reduction in cell viability exerted by this radiation at LSR (Figure 4d). It is likely that a suitable photothermal insult is required to trigger necroptosis in MCF7, but not sufficient to sustain such program after cell rescue. 

As expected, a different expression pattern was observed in response to laser insults on MCF7 incubated with CDs-PEG-BT/IT. The combination of hyperthermia and on demand IT release yield mRNA expression profiles in LSR resembling those observed in the ESR to the same experimental conditions. In particular, RT-qPCR analysis revealed that the co-occurrence of irinotecan-related DNA damages and hyperthermia enhanced the upregulation of necroptotic pathway (*BCL2L11, TNFα, MAP1LC3A,* and *RIPK1*). Thus, it appears that in contrast with LSR observed for the bare hyperthermia, combining photothermal stress and irinotecan release, a sustained cell death response occurred even after recovery of cells.

The overall results herein presented provide insights in the activation of cell death mechanisms triggered by CDs-PEG-BT/IT with/without NIR activation in MCF7 and MDA-MB-231 cells, which are known to exhibit significant phenotypic and genotypic differences. The upregulation in proapoptotic genes as well as the concurrent reduction in mRNA levels of prosurvival factors supports the activation of caspase-dependent cell death in MDA-MB-231 cells. Additionally, photothermal treatments boosted the antitumor effect on MDA-MB-231 promoting both apoptosis and necroptosis. The different MCF7 phenotypic/genotypic background, which includes epigenetic events disabling P53 activity [46] as well as the inactivation of caspase-3 [39], resulted in peculiar gene expression response. These cells are refractory to apoptosis, therefore, the activation of caspase-independent cell death mechanisms occurring via the activation of TNFα and RIPK1 pathway is not surprising.

## 3. Materials and Methods 

### 3.1. Chemicals

Urea (99%), citric acid (99.5%), N-(3-dimethylaminopropyl)-N’-ethylcarbodiimide hydrochloride (EDC) (99%), N-hydroxysuccinimide (NHS) (98%), ethanol (99.9%), phosphate buffered saline (PBS) pH 7.4, anhydrous N,N-dimethylformamide (DMF), rhodamine (99.5%), copper (II) sulfate (99%), ascorbic acid (99.5%), azide-PEG300-biotin, irinotecan hydrochloride (IT), 4-Pentynoic acid, and Sephadex G10, G15, and G25, dialysis tubing MWCO 2 kDa were purchased from Sigma Aldrich and used as received. NH2-PEG2000-CC was obtained as previously described [29].

MCF-7 and MDA-MB-231 cell lines were purchased from Sigma Aldrich and cultured in supplemented Dulbecco’s Minimum Essential Medium (DMEM) supplemented with 10% fetal bovine serum (FBS, EuroClone, Milan, Italy), 1% of penicillin/streptomycin (10,000 U mL^−1^ and 10 mg mL^−1^ respectively, EuroClone), and 1% of l-glutamine (EuroClone), at 37 °C in 5% CO_2_ humidified atmosphere. Cell Titer 96 Aqueous One Solution Cell Proliferation assay (MTS solution) was purchased from Promega (Madison, WI, USA).

### 3.2. Synthesis of Biotinylated Carbon Nanodots (CDs-PEG-BT)

CDs-PEG-BT were synthesized as described in detail in our previous work [29]. Briefly, naked CDs were prepared dissolving urea (6 g) and citric acid (3 g) in anhydrous DMF (30 mL). The reaction was conducted under solvothermal condition at 160 °C for 4 h. The product was precipitated in ethanol, retrieved by centrifugation, and dissolved in ultrapure water by sonication. Red-emitting CDs endowed with NIR-sensitive photothermal property were selected by size exclusion chromatography (SEC) combining G25, G15, and G10 packed in gradient mode [26]. Biotinylated CDs (CDs-PEG-BT) were prepared from red-emitting CDs by two steps of surface passivation [29]. In a first step, CDs (20 mg mL^−1^) were dissolved in ultrapure water (10 mL) and NH2-PEG2000-CC (250 mg) were added. EDC (24.92 mg, 0.13 mmol) and NHS (14.96 mg, 0.13 mmol) were then put into the reaction mixture at once and the pH was adjusted to 6.4. After 18 h, the crude reaction was purified by dialysis to give rise to a dark powder after freeze-drying. In the second step, 50 mg of both CDs-PEG-CC and biotin-PEG_3_-N_3_ were dissolved in 4 mL of ultrapure water by sonication. The reaction was carried out in presence of ascorbic acid (10%) and copper (II) sulfate (10%) as catalysts under nitrogen atmosphere with stirring for 18 h. The pure product (CDs-PEG-BT) was obtained as purplish powder after purification using SEC as described above. Yield: 88%.

### 3.3. Physiochemical Characterization of CDs-PEG-BT

The size distribution of the CDs-PEG-BT was characterized by atomic force microscopy (AFM). Precisely, 10 µL of the sample (0.1 mg L^−1^) was deposited on a mica substrate and dried in vacuum (10 mbar). AFM measurements were conducted in soft tapping mode through a Bruker FAST-SCAN microscope. The diameter distribution was assessed on the basis of their height from the AFM images. The structural characterization was attained by high-resolution transmission electron microscopy (HR-TEM) using a JEOL JEMS-2100 HR-TEM at 200 kV electron energy. The water dispersion of biotinylated CDs (1 mg L^−1^) was deposited on a 400 µm mesh Cu-grid covered by a holey amorphous carbon film, with nominal thickness of 3 nm. The lattice space (d-spacing) was calculated by inverse Fourier transform (IFFT) and was in agreement with Bragg’s law. The surface functional groups of CDs-PEG-BT was studied by FTIR spectroscopy using a Bruker Alpha II spectrometer (Billerica, MA, USA). Spectra of CDs-PEG-BT and the parent compound were obtained using a tablet of anhydrous KBr (≈0.2% w/w) and measures were recorded within the range of 400–4000 cm^−1^ (16 scans, resolution of 4 cm^−1^).

### 3.4. Photothermal Effect of the CDs-PEG-BT

The NIR photothermal conversion of the CDs-PEG-BT nanoheaters was evaluated monitoring the temperature change of a dispersion of CDs-PEG-BT in PBS pH 7.4 (200 µL, 0.5 mg mL^−1^) placed in a 96 multiwell irradiated with an 810 nm laser diode (power 2 W cm^−2^). The sample temperature was registered by a CEM Discover SP optical thermometer (±0.2 °C). PBS pH 7.4 (200 µL) was used as control.

### 3.5. Optical Characterization of CDs-PEG-BT

The absorption spectra of CDs-PEG-BT were obtained by double beam spectrophotometer (Shimadzu 2401PC UV-VIS). Steady-state absorption measurements were recorded in the 200–850 nm range. The absorption measurements of the aqueous dispersion of biotinylated CDs (0.1 mg mL^−1^) were conducted in a 1 cm quartz cuvette and compared with the virgin CDs. 

The fluorescence spectra of the CDs-PEG-BT and the plane CDs were obtained by a Shimadzu RF-5301(PC)S (Kyoto, Japan). The emission spectra of samples in the range of 560–850 nm were recorded by exciting at 540 nm. The quantum yield (QY) was assessed using rhodamine as a standard fluorescent compound in water at pH 13. 

### 3.6. Preparation of the Irinotecan-Loaded CDs-PEG-BT (CDs-PEG-BT/IT)

Irinotecan-loaded CDs-PEG-BT (CDs-PEG-BT/IT) were obtained by adsorption of irinotecan hydrochloride (IT) (15 mg) on the dot’s (20 mg in 3 mL water) shell by sonication (15 min × 2). After 16 h of incubation at 25 °C, the free drug was removed by dialysis (2 kDa), and the drug loading was calculated spectrophotometrically measuring the absorbance of the waste water at 366 nm and comparing the absorbance with that of a standard calibration curve of IT in water. Drug loading (DL) on a weight basis: 23.7%.

### 3.7. Drug Release Studies of the CDs-PEG-BT/IT

Either CDs-PEG-BT/IT (0.42 mg) or equivalent amount of free IT (0.1 mg) was dissolved in PBS pH 7.4 (1 mL) and placed into a dialysis tubing (2 kDa) against PBS pH 7.4 (9 mL). The dialysis was incubated in orbital shaker at 37 °C and 100 rpm for 48 h. During this time, at defined set times the external medium (0.2 mL) was withdrawn and replaced with equal volume of fresh medium. The amount of IT released was established spectrophotometrically as described above, and drug release kinetic was obtained as function of the incubation time. 

In a second set of experiments, the photothermal-triggered drug release was assessed treating the same dispersion of CDs-PEG-BT/IT in PBS pH 7.4 for either 50 or 200 s with an 810 nm laser (Gbox 15A/B by GIGAA Laser, Wuhan, China; power of 2 W cm^−2^ = 0.1 W mm^−3^) after 2, 8, and 20 h from the incubation. The release kinetic was obtained as above described, and the results were compared with the untreated control. Data shown as mean ± s.e.m. (*n* = 3; CV% < 3%)

### 3.8. Biological Characterization

#### 3.8.1. In Vitro Cytocompatibility Studies

The cytocompatibility of CDs-PEG-BT was established on human breast cancer cell lines (MCF-7, MDA-MB-231 by Sigma Aldrich, Milan, Italy) by the MTS assay (Promega, Madison, WI, USA). Cells were seeded in a 96-multiwell plate (1 × 10^4^ cells/well) and grown in supplemented Dulbecco’s Minimum Essential Medium (DMEM) as described above. After 24 h, the medium was replaced with fresh medium (200 µL) containing increasing amount of CDs-PEG-BT up to 10 mg mL^−1^. Untreated cells were used as negative control. Cell viability was assessed by MTS assay after either 24 or 48 h incubation. Briefly, the medium was replaced with PBS and cells were washed up with fresh PBS (200 µL). Then, 100 µL of DMEM and 20 µL of an MTS solution were added to each well and cells were incubated for 2 h at 37 °C and 5% of CO_2_ before measuring the absorbance at 492 nm using an Eppendorf AG AF2200 microplate reader (, Hamburg, Germany). Cells viability correspond to its percentage reduction against control cells. All independent experiments (*n* = 2) were performed in triplicates. 

The cytotoxicity of CDs-PEG-BT/IT was evaluated as above described for the drug-free carbon nanodots, but with qualifications. In particular, either CDs-PEG-BT/IT or free IT at a drug concentration per well ranging from 25 to 150 µg mL^−1^ was employed. Untreated cells were used as negative control. All culture experiments were performed in triplicates.

#### 3.8.2. NIR-Triggered Photothermal Cell Ablation In Vitro

The photothermal-triggered anticancer effect of CDs-PEG-BT and CDs-PEG-BT/IT was assessed on both MCF-7 and MDA-MB-231 cell cultures. Cells were seeded in a 96-multiwell plate as described above and, after an incubation time of 24 h, the medium was replaced with a dispersion of CDs-PEG-BT/IT in DMEM at the IC_50_ previously calculated for both cell lines (590 µg mL^−1^ ≈ 140 μg mL^−1^ of free IT). After that two different experimental sets were carried out to establish the Early Stage Response (ESR) and Long Stage Response (LSR), respectively. In the first one (ESR), cells were incubated for 23.5 h and treated with an 810 nm laser diode (power of 2 W cm^−2^) for either 50 or 200 s. After that, cells were incubated for 30 min and MTS assay was performed as described above. In a second set of experiments (LSR), cells were incubated for 4 h and treated with an 810 nm laser diode (power of 2 W cm^−2^) for either 50 or 200 s. Treated cells were then incubated for 20 h before performing the MTS assay as above. For all experiments, untreated cells were used as negative control and results were compared with those obtained with a dispersion of the empty CDs-PEG-BT. Statistical analysis was carried out on data of two independent experiments performed in triplicates.

#### 3.8.3. In Vitro Cell Uptake Study on MCF-7 and MDA-MB-231 Cells

Cell uptake of CDs-PEG-BT was evaluated by fluorescent microscopy (Zeiss “AXIO Vert. A1” Microscope Inverted, Oberkochen, Germany). Either MCF-7 or MDA-MB-231 cells were seeded at a density of 3 × 10^4^ cells/well into 8 well plate and cultured for 24 h. Then, the medium was replaced with a dispersion of CDs-PEG-BT in DMEM (500 µL; 0.5 mg mL^−1^) and cells were incubated for 4 h. After that, the medium was removed, the cell monolayer was washed twice with PBS pH 7.4 and the nuclei were stained with 4′,6-diamidino-2-phenylindole (DAPI). Images were recorded by a fluorescence microscope using a Zeiss Axio Cam MRm (Oberkochen, Germany). Untreated cells were used as negative control to set the autofluorescence.

#### 3.8.4. RNA Extraction and First-Strand cDNA Synthesis

Total RNA was extracted from control and treated cells (5.000 cells as starting material) using TRIzol Reagent (Invitrogen Corporation, Carlsbad, CA, USA) and following the manufacturer’s instructions. RNA was stored at −80 °C for future use. Purified RNA was treated with Deoxyribonuclease I, Amplification Grade (Sigma-Aldrich, Milan, Italy) to remove any residual genomic DNA contamination, and DNase I was inactivated by adding 50 mM EDTA. First strand cDNA was synthesized from each entire DNase I-treated RNA samples (about 50 ng) using the High-Capacity cDNA Reverse Transcription Kit (Thermo Fischer Scientific, Carlsbad, CA, USA) following the manufacturer’s instructions. The cDNA mixture was tested by PCR using *18S*, *GAPDH,* and *ACTB* primers (Table S1) and diluted 1:10 prior to use in Real-Time qPCR experiments.

#### 3.8.5. Gene Expression by Real-Time Quantitative Polymerase Chain Reaction (qPCR)

The qPCRs were performed using the BIO-RAD CFX96 system with BrightGreen 5X qPCR MasterMix (Applied Biological Materials Inc, Richmond, BC, Canada) as detection chemistry. The *18S* ribosomal RNA, *ACTB,* and *GAPDH* genes were selected as control genes based on their expression stability in all tested conditions. A normalization factor was calculated based on geometric averaging of the expression level of these reference genes and was used to quantify the expression levels of the target genes [60]. Serial dilutions of pooled cDNAs from both control and treated samples were prepared to determine the PCR efficiency of the target and reference genes (data not shown), and amplification efficiency ranged from 1.8 to 2.1. Primer sequences used in this study are listed in Table S1. Quantitative real-time PCRs were conducted according to the manufacturer’s recommended procedures, and each reaction was repeated in triplicate. The amplification conditions were the following: initial denaturation at 95 °C for 10 min and 40 cycles of 95 °C for 30 s and 60 °C for 50 s, followed by a melting curve from 60 °C to 95 °C. Amplicons were detected by agarose gel analysis after each PCR to confirm the amplification of the specific gene.

#### 3.8.6. Statistical Analysis

Experiments were performed in triplicate. Gene expression results presented as heat maps were generated via Heatmapper (available at http://heatmapper.ca/expression/). The mRNA levels are represented as mean centered, whereas standard deviation (SD) (*n* = 3) was below 0.5%. The results in bar plot were expressed as a mean value ± SD. Significant differences between values of different treated groups and the reference control groups were determined by *t*-test using Statistica 6.0 (StatSoft, Tulsa, OK, USA). *p* values less than 0.05 were considered statistically significant (Tables S2 and S3).

## 4. Conclusions

Approaches combining NIR–triggered photothermal treatments based on luminescent carbon nanodots and canonical anticancer drugs may overcome specific resistance to apoptosis eliciting alternative RCD routes such as necroptosis. Synergistic activation of multiple cell death pathways represents an interesting therapeutic strategy aiming at targeting resistant cancer cells in a more precise way. Herein, the anticancer theranostic activity of irinotecan-loaded red luminescent CDs bearing biotin as targeting pendants by means of discrete PEGylation, named “CDs-PEG-BT/IT,” against different resistant breast cancer cells was investigated. CDs-PEG-BT/IT released local heat and their drug payload in a NIR-light triggered on-demand fashion, implying effective anticancer effects after applying proper 810 nm photothermal treatments. It was proven that CDs-PEG-BT/IT can efficiently enter breast cancer cells displaying self-tracking ability in fluorescence imaging. A deep analysis of the gene expression pathways involved in RCD provides insights about the marked biocompatibility of bare CDs-PEG-BT vehicles. Moreover, the concurrent upregulation of proapoptotic genes and the downregulation of antiapoptotic genes, including some BCL2 family members, support the activation of the apoptotic event at least in MDA-MB-231 cells. Additionally, analysis of the gene expression signatures indicates that necroptosis can be induced only following CDs-PEG-BT/IT photothermal activation. Besides, photothermal activation of CDs-PEG-BT/IT implies both necroptosis and apoptosis by the TNFα and RIPK1 pathways thus ensuring synergistic and strong anticancer effects also in cells refractory to apoptosis as in the MCF7. On the whole, these data indicate that CDs-PEG-BT/IT is a safe and potentially effective candidate as a theranostic agent in IG-PTT of breast cancer.

## Supplementary Materials

The following are available online at https://www.mdpi.com/2072-6694/12/11/3114/s1, Table S1: Genes and oligonucleotide primers used in this study, Table S2: Statistical analyses by Student’s *t*-tests of RT-qPCR analyses in MCF7 cell line, Table S3: Statistical analyses by Student’s *t*-tests of RT-qPCR analyses in MCF7 cell line.
