# Supplementary material for: Carbon Nanodots for On Demand Chemophotothermal Therapy Combination to Elicit Necroptosis: Overcoming Apoptosis Resistance in Breast Cancer Cell Lines
Source: Cancers (Basel). 2020 Oct 25;12(11):3114. doi: 10.3390/cancers12113114 (PMC7692354; doi:10.3390/cancers12113114)
Supplement: Supplementary file 1 [file cancers-12-03114-s001.pdf]

Supplementary Materials: Carbon Nanodots for On Demand Chemo-photothermal Therapy Combination to Elicit Necroptosis: Overcoming Apoptosis Resistance in Breast Cancer Cell Lines

Aldo Nicosia, Gennara Cavallaro, Salvatore Costa, Mara Andrea Utzeri, Angela Cuttitta, Gaetano Giammona and Nicolò Mauro

Table S1. and oligonucleotide primers used in this study.

Gene name	Gene symbol	Accession Number	Primer sequence (5′ – 3′)
Apoptotic peptidase activating factor 1	*APAF1*	NM_001160	GCCAAGCAGGAGGTCGATAATG<br>GACCATCCTCAGAAAAGCAGGC
ATG12 autophagy related 12 homolog	*ATG12*	NM_004707	GGGAAGGACTTACGGATGTCTC<br>AGGAGTGTCTCCCACAGCCTTT
ATG3 autophagy related 3 homolog	*ATG3*	NM_022488	ACTGATGCTGGCGGTGAAGATG<br>GTGCTCAACTGTTAAAGGCTGCC
ATG5 autophagy related 5 homolog	*ATG5*	NM_004849	GCAGATGGACAGTTGCACACAC<br>GAGGTGTTTCCAACATTGGCTCA
ATG7 autophagy related 7 homolog	*ATG7*	NM_006395	CGTTGCCCACAGCATCATCTTC<br>CACTGAGGTTCACCATCCTTGG
BCL2-associated X protein	*BAX*	NM_004324	TCAGGATGCGTCCACCAAGAAG<br>TGTGTCCACGGCGGCAATCATC
B-cell CLL/lymphoma 2	*BCL2*	NM_000633	ATCGCCCTGTGGATGACTGAGT<br>GCCAGGAGAAATCAAACAGAGGC
BCL2-related protein A1	*BCL2A1 (BFL1)*	NM_004049	GGATAAGGCAAAACGGAGGCTG<br>CAGTATTGCTTCAGGAGAGATAGC
BCL2-like 1	*BCL2L1 (BCLXL)*	NM_138578	GCCACTTACCTGAATGACCACC<br>AACCAGCGGTTGAAGCGTTCCT
BCL2-like 11 (apoptosis facilitator)	*BCL2L11*	NM_006538	CAAGAGTTGCGGCGTATTGGAG<br>ACACCAGGCGGACAATGTAACG
Beclin 1, autophagy related	*BECN1*	NM_003766	CTGGACACTCAGCTCAACGTCA<br>CTCTAGTGCCAGCTCCTTTAGC
Bcl2 modifying factor	*BMF*	NM_033503	CAGTGGCAACATCAAGCAGAGG<br>GCAAGGTTGTGCAGGAAGAGGA
Caspase 2, apoptosis-related cysteine peptidase	*CASP2*	NM_032982	TGCCTTCTGTGAAGCACTGAGG<br>CGGAAAAGGGAGACTCAAGTCG
Caspase 3, apoptosis-related cysteine peptidase	*CASP3*	NM_004346	GGAAGCGAATCAATGGACTCTGG<br>GCATCGACATCTGTACCAGACC
Caspase 7, apoptosis-related cysteine peptidase	*CASP7*	NM_001227	CGGAACAGACAAAGATGCCGAG<br>AGGCGGCATTTGTATGGTCCTC
Caspase 9, apoptosis-related cysteine peptidase	*CASP9*	NM_001229	GTTTGAGGACCTTCGACCAGCT<br>CAACGTACCAGGAGCCACTCTT
COMM domain containing 4	*COMMD4*	NM_017828	CAGTGCTGAGTTTCATCCTCTCC<br>TGCTTCTCCTCATAACAGCGGC
Cylindromatosis (turban tumor syndrome)	*CYLD*	NM_015247	GGTAATCCGTTGGATCGGTCAG<br>AGTGCCTCTGAAGGTTCCATCC
Eukaryotic translation initiation factor 5B	*EIF5B*	NM_015904	AACGGAGGATTGAGAAACGGCG<br>TTCCCTGTGTCCACATGCCCAA
Fas (TNF receptor superfamily, member 6)	*FAS(TNFRSF6)*	NM_000043	GGACCCAGAATACCAAGTGCAG<br>GTTGCTGGTGAGTGTGCATTCC
UDP-N-acetyl-alpha-D-galactosamine:polypeptide	*GALNT5*	NM_014568	CCAGTGGATAGAGCCATTGAAGA<br>TCTCAGGAGAGTGGACCACACT

N-acetylgalactosaminyltransferase 5 (GalNAc-T5)			
Microtubule-associated protein 1 light chain 3 alpha	*MAP1LC3A*	NM_181509	GCTACAAGGGTGAGAAGCAGCT<br>CTGGTTCACCAGCAGGAAGAAG
Myeloid cell leukemia sequence 1 (BCL2-related)	*MCL1*	NM_021960	CCAAGAAAGCTGCATCGAACCAT<br>CAGCACATTCCTGATGCCACCT
Poly (ADP-ribose) polymerase 1	*PARP1(ADPRT1)*	NM_001618	CCAAGCCAGTTCAGGACCTCAT<br>GGATCTGCCTTTTGCTCAGCTTC
Poly (ADP-ribose) polymerase 2	*PARP2*	NM_005484	GGTGGCTTGTTCAGGCAATCTC<br>GGTGGCATAGTCCATCTGTAGC
Phosphoinositide-3-kinase, class 3	*PIK3C3(VPS34)*	NM_002647	GCGTTCTTTGCTGGCTGCACAA<br>CTCCAAGCAATGCCTGTAGTCTC
RAB25, member RAS oncogene family	*RAB25*	NM_020387	ACTGCTCTTCCTGGAGACCTCA<br>GCTGTTCTGTCTCTGCTTGGAC
Transmembrane protein 57	*TMEM57*	NM_018202	CTGAGCAGGAAGCCCGAAGTTT<br>CGATTCCGTAAGGTTTCGGTGC
Tumor necrosis factor	*TNFα*	NM_000594	CTCTTCTGCCTGCTGCACTTTG<br>ATGGGCTACAGGCTTGTCACTC
Human receptor (TNFRSF)-interacting serine-threonine kinase 1	*RIPK1*	NM_003804	TATCCCAGTGCCTGAGACCAAC<br>GTAGGCTCCAATCTGAATGCCAG
(Myc-DDK-tagged)-Human solute carrier family 25	*SLC25A4*	NM_001151	GCTGCCTACTTCGGAGTCTATG<br>TGCGACTGCCGTCACACTCTG
beta Actin	*ACTB*	NM_001101	CACCATTGGCAATGAGCGGTTC<br>AGGTCTTTGCGGATGTCCACGT
glyceraldehyde-3-phosphate dehydrogenase	*GAPDH*	NM_002046	GTCTCCTCTGACTTCAACAGCG<br>ACCACCCTGTTGCTGTAGCCAA
18S ribosomal RNA	*18S*	M10098.1	CGGCTACCACATCCAAGGAA<br>CTGGAATTACCGCGGCT

Table S2. Statistical analyses by t Student's of RT-qPCR analyses in MDA-MB-231 cell line, p values less than 0.05 are indicated by * and considered statistically significant.

			**ESR NIR_{50}**		**ESR NIR_{200}**		**LSR NIR_{50}**		**LSR NIR_{200}**	
	**CDs-PEG-BT**	**CDs-PEG-BT/IT**	**CDs-PEG-BT**	**CDs-PEG-BT/IT**	**CDs-PEG-BT**	**CDs-PEG-BT/IT**	**CDs-PEG-BT**	**CDs-PEG-BT/IT**	**CDs-PEG-BT**	**CDs-PEG-BT/IT**
APAF1				*	*			*		
ATG12		*	*	*	*	*	*	*	*	*
ATG3				*	*		*			
ATG5		*	*		*		*	*		*
ATG7		*	*	*		*	*	*		*
BAX				*		*				*
BCL2		*	*	*	*	*	*	*	*	*
BCL2A1		*	*	*	*	*	*			*
BCL2L1		*					*		*	*
BCL2L11	*	*	*	*	*	*	*	*	*	*
BECN1	*		*	*	*	*	*	*	*	*
BMF		*		*		*		*		*
CASP2							*		*	*

CASP3		*		*	*	*	*	*	*	*
CASP7		*		*		*	*	*	*	*
CASP9				*		*				
COMMD4		*							*	
CYLD		*		*	*	*				*
EIF5B		*			*				*	*
FAS				*		*				
GALNT5	*	*	*	*	*	*	*	*	*	*
MAP1LC3A		*		*			*	*		*
MCL1		*	*	*	*	*	*	*	*	*
PARP1									*	*
PARP2						*			*	
PIK3C3		*			*			*		
RAB25									*	
TMEM57		*	*	*	*	*	*	*	*	*
TNFα		*		*		*		*		*
RIPK1			*	*	*	*	*	*	*	*
SLC25A4						*			*	

Table S3. Statistical analyses by t Student's of RT-qPCR analyses in MCF7 cell line, *p* values less than 0.05 are indicated by * and considered statistically significant.

			**ESR NIR50**		**ESR NIR200**		**LSR NIR50**		**LSR NIR*200***	
	**CDs-PEG-BT**	**CDs-PEG-BT/IT**	**CDs-PEG-BT**	**CDs-PEG-BT/IT**	**CDs-PEG-BT**	**CDs-PEG-BT/IT**	**CDs-PEG-BT**	**CDs-PEG-BT/IT**	**CDs-PEG-BT**	**CDs-PEG-BT/IT**
APAF1	*	*		*	*		*	*	*	*
ATG12					*	*				
ATG3		*		*	*			*		*
ATG5	*	*		*	*	*	*	*		*
ATG7	*	*		*	*	*	*	*		*
BAX	*				*	*	*	*		*
BCL2	*	*	*	*	*		*		*	*
BCL2A1	*				*	*	*	*		
BCL2L1					*		*			
BCL2L11		*		*	*	*	*	*	*	*
BECN1	*	*	*	*	*	*	*	*	*	*
BMF										
CASP2		*		*	*		*	*		*
CASP3	*	*	*	*		*		*		*
CASP7	*	*	*	*	*	*	*	*	*	*
CASP9	*		*	*		*			*	*
COMMD4					*					
CYLD	*	*	*	*		*		*	*	*
EIF5B	*	*		*	*	*	*	*		*
FAS	*		*			*		*	*	
GALNT5	*	*	*	*	*	*	*	*	*	*

MAP1LC3A				*	*	*		*		*
MCL1		*		*	*	*	*	*		*
PARP1	*	*		*	*	*		*		*
PARP2		*		*		*	*	*		*
PIK3C3	*	*	*	*		*	*	*	*	*
RAB25		*		*	*			*		*
TMEM57		*		*	*			*		*
TNFα	*	*	*	*	*	*	*	*	*	*
RIPK1	*	*		*	*	*		*	*	*
SLC25A4	*	*	*	*			*	*	*	*
